# Investigating dysbiosis and microbial treatment strategies in inflammatory bowel disease based on two modified Koch’s postulates

**DOI:** 10.3389/fmed.2022.1023896

**Published:** 2022-11-10

**Authors:** HanZheng Zhao, WenHui Zhang, Die Cheng, LiuPing You, YueNan Huang, YanJie Lu

**Affiliations:** ^1^Department of General Surgery, The Second Affiliated Hospital of Harbin Medical University, Harbin, China; ^2^Department of Pain Medicine, Harbin Medical University Cancer Hospital, Harbin, China; ^3^Cancer Research Laboratory, Chengde Medical College, Chengde, China

**Keywords:** Koch’s postulates, inflammatory bowel disease, dysbiosis, microbial treatment, gut microbiome

## Abstract

Inflammatory bowel disease (IBD) is a chronic non-specific inflammatory disease that occurs in the intestinal tract. It is mainly divided into two subtypes, i.e., the Crohn’s disease (CD) and ulcerative colitis (UC). At present, its pathogenesis has not been fully elucidated, but it has been generally believed that the environment, immune disorders, genetic susceptibility, and intestinal microbes are the main factors for the disease pathogenesis. With the development of the sequencing technology, microbial factors have received more and more attention. The gut microbiota is in a state of precise balance with the host, in which the host immune system is tolerant to immunogenic antigens produced by gut commensal microbes. In IBD patients, changes in the balance between pathogenic microorganisms and commensal microbes lead to changes in the composition and diversity of gut microbes, and the balance between microorganisms and the host would be disrupted. This new state is defined as dysbiosis. It has been confirmed, in both clinical and experimental settings, that dysbiosis plays an important role in the occurrence and development of IBD, but the causal relationship between dysbiosis and inflammation has not been elucidated. On the other hand, as a classic research method for pathogen identification, the Koch’s postulates sets the standard for verifying the role of pathogens in disease. With the further acknowledgment of the disease pathogenesis, it is realized that the traditional Koch’s postulates is not applicable to the etiology research (determination) of infectious diseases. Thus, many researchers have carried out more comprehensive and complex elaboration of Koch’s postulates to help people better understand and explain disease pathogenesis through the improved Koch’s postulates. Therefore, focusing on the new perspective of the improved Koch’s postulates is of great significance for deeply understanding the relationship between dysbiosis and IBD. This article has reviewed the studies on dysbiosis in IBD, the use of microbial agents in the treatment of IBD, and their relationship to the modified Koch’s postulates.

## Introduction

Inflammatory bowel disease (IBD) is a chronic non-specific inflammatory disease that occurs in the intestinal tract. Patients with IBD often present with abdominal pain, diarrhea, fever, nutritional disorders, and weight loss, as well as other recurrent clinical symptoms. IBD is divided into two subtypes, i.e., the Crohn’s disease (CD) and ulcerative colitis (UC). The difference between UC and CD lies in that the UC lesions mainly involve the colon, and the lesions are limited to the superficial part of the large intestine and have a continuous distribution; while CD involves all segments of the digestive tract, mainly in the terminal ileum and adjacent colon (1). IBD patients also have many extra-intestinal manifestations and complications, such as peripheral arthropathy, erythema nodosum, primary sclerosing cholangitis, nephrolithiasis, peripheral neuritis, and anemia. It has the characteristics of systemic diseases (2). The incidence of IBD is higher in western countries, which might be related to urbanization, industrialization, and adjustment of dietary structure. In recent years, globalization has led to rapid economic development of emerging industrial countries such as China. With the society modernization, the incidence of IBD has increased. The incidence of IBD has risen sharply (3), although its incidence is still significantly different in different countries and regions. However, with the passage of time, the incidence of IBD has shown an upward trend in different regions, making IBD gradually known as a global public health problem (4). The pathogenesis of IBD has not yet been elucidated (3). Currently, it is believed that the environment (5), immune disorders (6, 7), genetic susceptibility (8), and gut microbes (9) represent the main factors for the disease pathogenesis. A chronic inflammatory state would result from disruption of the homeostasis between the microbiota, intestinal epithelial cells, and immune cells by genetic and environmental factors (e.g., antibiotics, smoking, and diet) (10).

With the development of the new-generation sequencing technologies and the gut microbiome macrogenomics programs, gut dysbiosis has been better understood. Studies have found that gut dysbiosis can cause and/or modulate most of the major pathogenic causes of IBD, such as the impaired intestinal epithelial cell function, impaired recognition of pathogenic bacteria, and abnormal innate immune responses (11, 12). Therefore, the gut microbiome has received more attention in recent years. It is currently believed that the dysbiosis is not only one of the pathogenic factors of IBD, but also may be the central factor of multifactorial pathogenesis.

**FIGURE 1:**
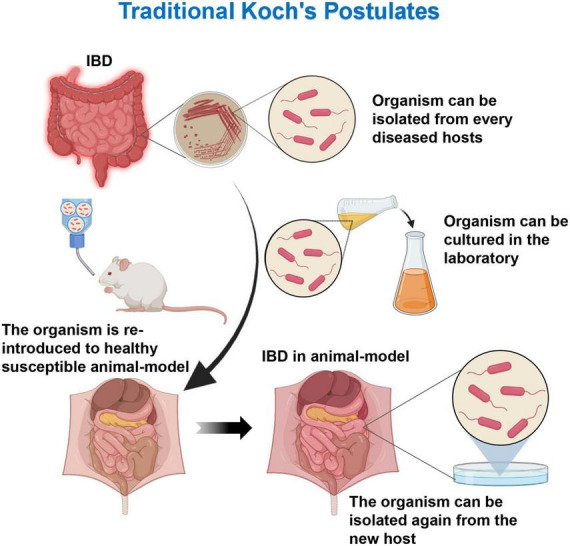
Traditional Koch’s postulates. The microorganism must be present in every case of the disease and can be isolated from the diseased host. After cultured in the laboratory, the microorganism can cause the same disease when introduced into a new host.

The traditional Koch’s postulates (one pathogen, one disease) requires the pathogen to meet the following criteria: (1) the microorganism must be present in all disease cases and not in healthy individuals; (2) the microorganism can be isolated in patients and be purified in the culture medium; (3) the pure cultured microorganism can inoculate a healthy susceptible host, which can lead to the recurrence of the disease; and (4) the microorganism can be isolated and cultured again in the test diseased host (Figure 1). The Koch’s postulates provides a standard for proving the role of organisms in disease. However, with the deepened understanding of diseases, the traditional Koch’s postulates has been unable to meet the etiological assumptions of some microorganisms and/or diseases. Therefore, the Koch’s postulates needs to be improved and modified. Dysbiosis is an ecosystem state that includes diverse microbes and their complex interactions, although many experiments suggest that individual microbes may play important roles in the immune regulation. However, it is currently believed that the gut microbiota dysregulation as a whole plays a key role in the persistent inflammatory response of chronic diseases (13, 14). Therefore, for such a complex group of pathogens, the classical Koch’s postulates might not be comprehensive enough. Through two improved Koch’s postulates, namely, the ecological and symbiotic Koch’s postulates, the dysbiosis and the onset of IBD and the re-establishment of the balance state would be linked with the disease remission. The etiology, pathogenesis, and improvement of the treatment plan have important guiding significance.

## Gut microflora and inflammatory bowel disease

### Symbiotic microflora

There are about 100 trillion microorganisms in the human gastrointestinal tract, including bacteria, viruses, fungi, archaea, and protists (15). The genetic catalog of the gut microbiome shows that more than 99% of them are bacteria (16). The diversity of bacteria increases from 15,000 to 36,000 species according to the rRNA sequence analysis (17). The concept of enterotype in terms of gut microflora has been proposed in recent studies, which identifies three enterotypes: Bacteroides enterotypes, Privobacterium enterotypes, and Ruminococcus enterotypes. All of these samples are pooled around the three enterotypes based on their propensity to make up the community, and the three enterotypes could be varied by the level of one of Bacteroidetes, Privotella, and Ruminococcus. Mutual identification and no correlation between enterotypes and distinct phenotypic characteristics (such as gender, age, race, and country) suggest that changes in the gut microbiome are stratified rather than being continuous (18). Most of the bacteria found in the adult gut belong to the genera Bacteroides, Parabacteroides, and Clostridium (19, 20). The human gut microbiota has co-evolved with the host through a symbiotic relationship. A healthy gut microbiota is important for nutrient uptake of the host, and the immune-microbiota pathway is associated with the maintenance of the homeostasis between mammalian feeding and weight (21). Commensal microbes play an important role in the development and maturation of the immune system (22). Gut microbes are also associated with diurnal fluctuations in host circadian transcription, physiology, and disease susceptibility (23). The microflora maintains the homeostasis of host physiology by competing with potential pathogens for nutrient sites, producing antimicrobial factors, and imposing colonization resistance to prevent the growth of potential pathogens.

### Dysbiosis in inflammatory bowel disease patients

The gut microbiome of healthy individuals is highly individualized (16, 19, 24). Although the microbiota differs in healthy individuals, this variation remains at the level of health plane (HP), whereas the microbiota in IBD patients fluctuates. Therefore, compared with healthy people, the gut microflora of IBD patients is in a state of dysregulation (25, 26), which is manifested by changes in the composition, diversity and stability of the microbiota (26–28). Beneficial or commensal bacteria (such as Bacteroidetes, Firmicutes, and Actinobacteria) are reduced in IBD patients, accompanied by increases in pathogenic species (such as Proteobacteria) (29, 30). The ratio of potentially pathogenic microbes to beneficial commensal microbes appears to play a key role in the disease development (31). Studies have shown that IBD patients have a substantial reduction in the gut Bifidobacterium and Lactobacillus (32), which are immunomodulatory bacteria. Bifidobacteria reduce intestinal pH by fermenting lactic acid, thereby preventing the colonization of pathogenic *Escherichia coli* (33, 34), while certain potentially pathogenic organisms (such as Klebsiella, Enterobacter, Proteus, and fungi) are increased in IBD patients (35). Certain *E. coli* genera, such as adherent-invasive *E. coli* and diffuse-adherent *E. coli*, have elevated proportions in patients with IBD, and patients with active CD have a greater proportion of adherent-invasive *E. coli* than controls (36), while most of the *E. coli* isolated from the feces of UC patients are diffuse-adherent *E. coli* (37). Studies have shown that intestinal flora imbalance is often related to the weakening of intestinal mucosal barrier function and the activation of inflammatory cells (38). Dysbiosis would change the proteome in the host, and affect the mitochondrial function, resulting in a pro-inflammatory state (39). In healthy individuals, the intestinal barrier consists of an intact layer of epithelial cells that are tightly connected by the claudin protein family, and the intestinal epithelial cells create a mucosal barrier that allows the microflora to interact with the host immune cell sequestration, thus reducing intestinal permeability. In patients with active CD, claudin-2 is upregulated, while claudin-5 and claudin-8 are downregulated and redistributed, resulting in a discontinuous state of tight junctions between epithelial cells (40, 41). Due to the existence of dysregulation, individuals have a greatly increased likelihood of opportunistic infection, resulting in low-grade inflammation of the mucosa, followed by increased intestinal permeability, ultimately leading to the so-called permeable gut (42, 43), i.e., the impaired intestinal barrier function. This would create a vicious circle, where intestinal barrier impairment will exacerbate intestinal inflammation and changes in the composition of the gut microbiome, eventually leading to systemic inflammation (44). Single-chain fatty acids (SCFAs), the most abundant microbial metabolites in the gut lumen, have been considered to be potential mediators of gut microbiota affecting gut immune function, which can regulate the expression of pro-inflammatory factors such as interleukin 6 (IL-6), IL-12, and tumor necrosis factor alpha by activating macrophages and dendritic cells, and thereby cause an anti-inflammatory effect of the immune system (45, 46). Under intestinal homeostatic conditions, peroxisome proliferator-activated receptor-γ (PPAR-γ), a nuclear receptor mainly synthesized in intestinal epithelial cells, is activated by butyrate. PPAR-γ promotes mitochondrial β-oxidation and oxidative phosphorylation of single-chain fatty acids in colonocytes to maintain a local hypoxic microenvironment. The obligate anaerobic SCFA-producing bacteria grow vigorously in such an environment. The colonization and growth of facultative anaerobic enteric pathogens are inhibited, and the facultative anaerobic Enterobacteriaceae are significantly increased when the intestinal microbiota is disturbed (47), leading to impaired anti-inflammatory effects of the host intestinal immune system. Experiments have found that wild mice co-bred with NOD2 gene-deficient mice have increased expression of apoptosis, necrosis, and oncogenes in their offspring, and the inflammation caused by chemically induced colitis would be more severe (48). At the same time, Torres et al. (49) have found that during pregnancy, pregnant women with IBD still have IBD-related microbial imbalances in the gut, and changed diversity and richness of bacteria in the neonatal gut. Neonatal dysbiosis microflora transplanted into germ-free mice would elicit abnormalities in the mouse gut immune system. This suggests that susceptibility to intestinal inflammatory disease may be due to the inheritance of a dysregulated microbiota that not only sensitizes the intestinal mucosa to chemical damage, but also triggers immune abnormalities in the gut. Impaired intestinal epithelial function, dysfunctional recognition of pathogens, and abnormal immune responses that accompany dysbiosis, would severely reduce intestinal resistance to pathogenic microbial colonization. The microbiota associated with IBD is rich in pathogenic bacteria. It is synergistic with pathogens in the process of exacerbating pathological reactions (50).

### Dysbiosis and ecological Koch’s postulates

**FIGURE 2:**
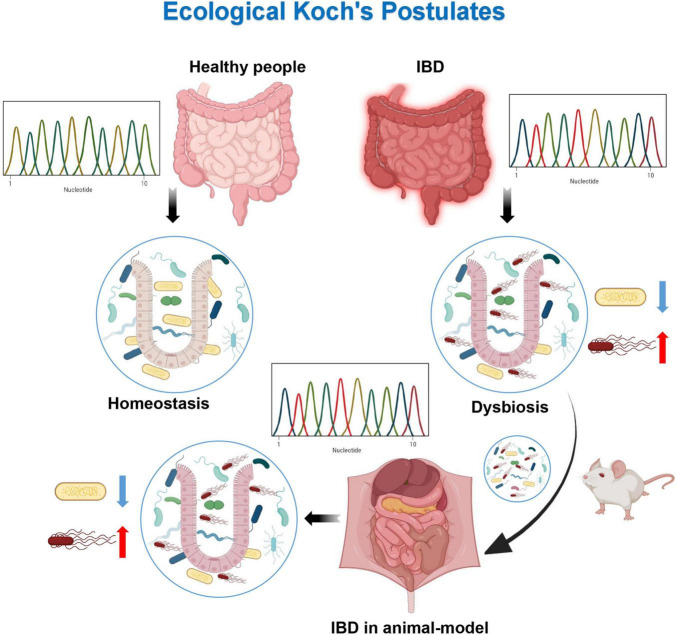
Ecological Koch’s postulates. By sequencing healthy individuals and diseased patients, it has been found that their microflora are different. Dysbiosis of microflora can be obtained from the diseased patients. The dysbiosis of microflora can cause the same disease when introduced into a healthy host. The dysbiosis of microflora can be detected from the diseased hosts.

Ecological Koch’s postulates (one gut ecosystem state, one disease): (1) an unbalanced microbiota with similar composition/characteristics would be found in all affected individuals; (2) an unbalanced microbiota can be obtained from a diseased host; (3) putting the obtained imbalanced flora into a sterile host and putting the host in a similar environment, and the host would produce similar symptoms; and (4) in a newly invaded host, the composition of the dysregulated microflora remains relatively stable (Figure 2) (51). Compared to the disease caused by a single pathogen, Koch’s postulates of ecology emphasizes the overall role of the microflora. The role of gut microflora dysbiosis in the pathogenesis of IBD has been investigated using the ecological Koch’s postulates. Just as the relationship between H. pylori and gastric ulcers satisfies the classical Koch’s postulates (52), this imbalance can be extracted to lead to the disease recurrence in healthy individuals. Experimental studies using mouse models of single bacteria related to IBD, such as *E. coli*, fecal *E. coli*, Bacteroidetes, etc., have shown that a single bacteria as a pathogen can induce chronic inflammation in susceptible hosts (53–55). However, a single bacterium does not reflect the complexity and interindividual variability of the gastrointestinal microflora. By combining seven human IBD-associated gut bacteria, a simplified bacterial consortium, SIHUMI, has been obtained. Eun et al. (56) have successfully colonized SIHUMI in experimental mice and have found that SIHUMI induces colitis in mice, in an antigen-specific manner. But this consortium is still too simplistic compared to the IBD microflora. Studies have reported that obesity-related metabolic phenotypes in humans can be transferred to recipient mice by microbiota transplantation (57). This suggests that the transfer of microflora can trigger related diseases, which provides ideas for IBD-related microflora transplantation experiments. Reinoso et al. (58) have shown that colonization of the microbiota transfer susceptibility to colitis in mice by means of fecal microbiota transplantation (FMT), making the incidence and severity of chronic colitis in recipients less than in donors. Schaubeck et al. (59) have reported that compared with experimental mice, germ-free mice do not develop CD-like ileitis, suggesting that the microbiota would be necessary for the development of intestinal inflammation. In addition, in this study, the disease-related dysbiosis flora is isolated from the cecum of the experimental mice, and the isolated disease-related dysbiosis flora of the cecum is introduced into the germ-free environment genetically susceptible mice, resulting in the latter CD-like gyrus, and occurrence of enteritis. It has been shown that CD-like ileitis can be transmitted through the dysbiosis of the microbiota, that is, the whole of microbiological dysbiosis as a pathogenic factor conforms to the second and third items of the Koch’s postulates. Subsequently, the cecal bacteria of the recipient mice are subjected to the high-throughput sequencing analysis, and it has been shown to be consistent with the donor flora composition. The dysregulated flora is isolated again in the newly pathogenic mice, which is in line with the fourth item of the Koch’s postulate. The results herein prove for the first time that there is a causal relationship between the disease-related flora imbalance and the occurrence of CD-like ileitis in the experimental state (59). These findings provide evidence and ideas for microbiological dysbiosis as a central factor in the pathogenesis of IBD.

Based on the experimental results of ecological Koch’s postulates above, we make a similar analogy, i.e., if the microecological imbalance can lead to the occurrence of IBD, then when we restore the balance of the microflora, the remission or even disappearance of the disease state would occur. In recent years, experimental advances to assess the therapeutic potential of the gut microbiota in the treatment of IBD support the hypothesis that host microbiota balance can be reconstituted, which would elicit clinical remission in IBD by administration of appropriate microbes.

## Microbial therapeutic strategies for inflammatory bowel disease

### Antibiotics

Experiments have shown that the application of antibiotics can induce remission in the acute phase of IBD and prevent the recurrence of clinical symptoms in IBD patients (60–63). Antibiotics can improve the microbial environment of IBD patients by reducing pro-inflammatory bacteria and increasing beneficial bacteria in the intestinal lumen of IBD patients (64). A meta-analysis has been performed on the role of antibiotics in the treatment of IBD, and consistent results have been found. The first report has suggested that antibiotics may be beneficial for the relief of clinical symptoms in UC and CD, and the second report supports that antibiotics can improve clinical outcomes in IBD (65, 66). Clinical trials have shown that the combined application of antibiotics is more effective in the treatment of IBD, such as OTICS (metronidazole, amoxicillin, doxycycline, and vancomycin) combination therapy in the clinical treatment of moderate to severe refractory ulcerative colitis in children, and satisfactory results have been obtained in the process (67). In addition, there are data showing that metronidazole, when used in combination with azithromycin, is effective in inducing remission in pediatric CD (68). Meanwhile, antibiotics application can adversely affect the gut microflora, and short-term use of antibiotics will lead to a decrease in antibiotic-sensitive bacteria, a decrease in the overall diversity of the flora, and an increase in the possibility of colonization by naturally resistant bacteria (69, 70).

### Prebiotics and probiotics

Prebiotics are non-digestible carbohydrates that are selectively fermented by gut bacteria and promote their activity, resulting in beneficial effects on the host (71). Prebiotics have established an important position in the treatment of IBD by selectively stimulating the growth of commensal microorganisms in the gut microflora and increasing the production of SCFAs, as highlighted in a recent meta-analysis. Beneficial effects in rats with TNBS-induced colitis include increased growth of Lactobacillus and Bifidobacterium, increased production of SCFAs, reduction of colon macroscopic lesions, and regression of inflammatory markers (72, 73). Probiotics are defined as live microorganisms that, when ingested in sufficient amounts, are beneficial to the health of the host. Common probiotics include Lactobacillus, Bifidobacterium, and Boula. In the treatment of IBD, the potential beneficial effects of probiotics include: (1) inhibition of pathogen invasion; (2) improvement of epithelial barrier function; and (3) immunomodulation (74). Clinical trials have shown that the mixed use of lactobacillus and VSL#3 (probiotic combination) shows high efficacy in the treatment of children with IBD, and VSL#3 can also effectively induce remission of active UC. Probiotics seem to be a safe alternative to 5-aminosalicylate as an effective agent for maintaining low activity of biologics in IBD (75, 76). However, to date, the ability to understand and monitor the microbiome in IBD is limited, and clinical trials related to probiotics have not been rationally designed to correct the microbial dysbiosis that may lead to IBD. Therefore, any findings of treatment effects should all be accidental. According to the modern microbial pathogenesis model of inflammatory bowel disease, it is necessary to apply macrogenome sequencing technology to identify specific strains with biologically credible efficacy in IBD, and to design further experiments to study targeted and specific probiotics.

### Fecal microbiota transplantation

Fecal microbiota transplantation is a treatment that injects the fecal microflora of a healthy donor into the recipient’s gut. Initially, FMT has been used as a clinical treatment method for the Clostridium difficile recurrent infection (CDI). In a landmark article, the first randomized controlled trial of FMT in the treatment of CDI has been published, and the results show that 81% of patients recover after FMT has been administered through a nasoduodenal tube, while the curing rate in the control group is lower than 31% (77). Thereafter, numerous experiments have demonstrated the role of FMT in the treatment of CDI, and it has been identified as a highly effective therapy for CDI (78), with the curing rate of as high as 97% (79). The application of FMT in CDI patients aims to restore the homeostasis of the patient’s gut microflora, and unlike treatments such as antibiotics and immunosuppressants, FMT significantly increases the number and diversity of recipient fecal bacterial populations. The longer engraftment period of bacterial populations (80, 81) would lead to further assumptions to evaluate the effectiveness of FMT in the treatment of other types of diseases in which dysbiosis is a major pathogenic factor affecting disease progression. Therefore, FMT has been expected to be a potentially promising treatment for IBD. Results of a double-blinded, randomized, and placebo-controlled trial in 81 subjects have shown that the application of high-dose, multi-donor FMT increases the microbial diversity of the experimental group, which persists and is effective in inducing activity clinical remission of stage UC (82). Another experiment, by randomly dividing the subjects into the experimental group (50 ml FMT enema from healthy donors) and control group (placebo, 50 ml water enema), conducts a randomized controlled trial, and the results show that compared with the control group, the experimental patients achieve stable primary efficacy indicators (Mayo score ≤ 2 and endoscopic Mayo score of 0), with no difference in adverse reactions between these two groups. The fecal microbial diversity of patients receiving FMT is significantly higher than the placebo group (*p* = 0.02, Mann–Whitney *U* test) (83). These findings show that FMT can be used as an effective clinical treatment for UC. An interesting phenomenon has been observed in the second group of experiments. The feces of one of the six donors induce UC remission in 39% of the experimental group patients, suggesting that FMT therapy may have a donor-receiver compatibility effect in IBD (83). Fang et al. (84) have conducted a meta-analysis of the therapeutic effect of FMT in IBD, and their findings confirm the effectiveness of FMT in the treatment of IBD patients. In addition, their findings suggest that the use of fresh or frozen donor stool, different routes of administration, and factors such as whether there is a history of antibiotic use, have no effects on the efficacy of FMT in the treatment of IBD patients. This suggests that FMT may be a potential rescue therapy for the treatment of acute exacerbations of IBD, or even as an initial standard treatment regimen. He et al. (85) investigated the microbiome changes before and after FMT in IBD patients. In their study, 3–5 Units of fresh fecal bacteria (1 U = 1 × 10^13^ cells) in suspension (1 U with 20 ml saline) was delivered to patient’s gut. They found that, after FMT, the diversity of the gut microbiota increased significantly in recipients, and approximated the level of healthy donors, indicating the ability of FMT to improve the diversity of disturbed microbiota. Moreover, the relative abundance of the dominant bacteria in recipients after FMT decreased toward the level of the donor, which illustrated the modulating effect of FMT on microbial community structure. In addition, the authors classified the gut bacteria in recipients after FMT into two categories, residents and colonizers. The residents were abundant in patients before FMT, and the colonizers were relatively absent in patients before FMT but newly acquired from donors. Then, they defined the ratio of colonizers to residents after FMT as C2R and found that C2R was significantly elevated in the FMT response group compared to the failure group, suggesting the successful colonization of more bacteria from the donor in the gut of the recipients. These results imply the importance of diversity changes before and after FMT and bacterial colonization in IBD patients, which was also find in CDI patients who are also characterized by gut dysbiosis (85).

In all studies, short-term use of FMT appears to be effective and safe, with most adverse effects being mild, self-limiting, and gastrointestinal (86). In theory, however, FMT therapy involves the introduction of an unspecified suspension of active microbiota that may cause bacterial-related diseases. Therefore, the main limitation of FMT is the long-term effect and safety issues (87, 88). The most common adverse reaction is abdominal pain, and even serious adverse reactions such as IBD recurrence, serious infection, or death may occur (87). The current treatment of IBD-related FMT is still in its infancy, and IBD is a complex disease involving multiple factors. Numerous results suggest that when combined with microbial profiling of donors and recipients, FMT may be an effective treatment and a powerful research tool, which will aid in the establishment of patient classification criteria and the development of personalized microbial therapy program. According to the latest ECCO guidelines, FMT is very promising for the treatment of active UC, and meanwhile, more researches (route of administration, donor characteristics, and frequency and duration of treatment) are needed to determine the optimal treatment of IBD protocol to improve the efficacy and safety of FMT in the disease treatment (89). In this section, we summarized some of the existing microbial therapeutic strategies in IBD patients, and the detailed study characteristics are provided in Table 1.

**TABLE 1:** The relationship between different aspects of microbial therapies.

Microbial therapy	Model	Intervention strategy	Results	References	Beneficial effects	Deficiency
Antibiotics	Human	Ciprofloxacin 500 mg, orally twice daily for 6 month	Experiment group has a significantly lower disease activity scores than placebo group	George et al. (60)	Pathobiont killing; population expansion of beneficial microorganisms	Inability to selectively eliminate pathobiont without potentially affecting normal microorganisms
	Human	Oral amoxicillin, tetracycline, and metronidazole for 2 weeks	63.3% of steroid refractory and 73.4% of steroid dependent patients showed a clinical response within 2 weeks	Kato et al. (61)		
	Human	Oral 800 mg of rifaximin twice a day for 12 weeks were compared with those from patients who received placebo	All the patients in experiment group were in remission after 12 weeks of treatment in comparison with 84% (70/83) of the placebo group, and the difference was also persistent at the 24-week follow-up	Jigaranu et al. (62)		
	Human	Metronidazole (0.6 g/d) for 12 month	Metronidazole was useful in the maintenance of remission in patients with UC	Gilat et al. (63)		
	Meta-analysis	/	Antibiotics was beneficial for the relief of clinical symptoms in UC and CD	Khan et al. (65)		
	Meta-analysis	/	Antibiotics can improve clinical outcomes in IBD	Wang et al. (66)		
	Human	Antibiotic cocktail [amoxicillin 50 mg/kg divided by 3 (up to 500 mg X3/d), metronidazole 5 mg/kg X3/d (up to 250 mg X3/d), and doxycycline 2 mg/kg X2/d (up to 100 mg X2/d)] for 3 weeks	Wide-spectrum antibiotic cocktail in Pediatric UC seems promising outcome in half of patients	Turner et al. (67)		
	Human	Azithromycin 7.5–10 mg/kg day up to a maximal dose of 500 mg, once daily, for five consecutive days per week for 4 weeks, and three times a week for the following 4 weeks in conjunction with metronidazole 15–20 mg/kg/day in two divided doses, given daily for 8 weeks	Azithromycin and metronidazole therapy may be effective in inducing clinical remission in mild-moderate luminal CD in children and young adults	Levine et al. (68)		
Prebiotics and probiotics	Rats	Administration of combination of FOS and resistant starch (37.5% FOS and 62.5% resistant starch) (2 g/rat/day) for 3 weeks	Increasing lactobacilli and bifidobacteria, improving the intestinal barrier function	Maria et al. (72)	Increasing population of beneficial microorganisms (such as Bifidobacterium and Lactobacillus) preventing the colonization of pathobiont (such as *E. coli*)	Limited effects on the overall composition of the microbiome as the inability to monitor the microbiome in IBD patients
	Rats	Administration of KOS (1.0 and 4.0 g/kg/day) for 2 weeks	Increased production of SCFAs, reduction of colon macroscopic lesions, and regression of inflammatory markers	Liu et al. (73)		
	Human	VSL#3 (probiotic combination) oral administration twice daily for 90 days	VSL#3 has anti-inflammatory effects and could reduce endoscopic recurrence after surgery for Crohn’s Disease	Fedorak et al. (75)		
	Human	VSL#3 (probiotic combination) oral administration twice daily for 6 weeks	Effectively induce remission of active UC	Bibiloni et al. (76)		
FMT	Human	50 ml FMT or placebo consisting of 50 ml water given as a retention enema once weekly for 6 weeks	Stool from patients receiving FMT had greater microbial diversity, compared with baseline, than that of patients given the placebo	Moayyedi et al. (83)	Restoring microbial diversity; population expansion of beneficial microorganisms	long-term effects and safety issues (side effect such as abdominal pain, severe infection, etc.)
	Human	3–5 Units of fresh fecal bacteria (1 U = 1 × 10^13^ cells) in suspension (1 U with 20 ml saline) was delivered through one of the three delivery ways: endoscopic, nasojejunal tube, or transendoscopic enteral tubing, Patients were assessed at the point of baseline, day 3, week 4, week 12, and every 3 months after each FMT	Diversity of the patient’s gut microbiota increased significantly in recipients, and approximated the level of healthy donors	He et al. (85)		
	Meta-analysis	/	FMT is an effective and safe therapy for both pediatric and adult IBD; fresh or frozen donor stool, delivery route, and antibiotic pretreatment or not have no impact on the efficacy of FMT in IBD	Fang et al. (84)		

### Microbial therapeutic strategies and commensal Koch’s postulates

**FIGURE 3:**
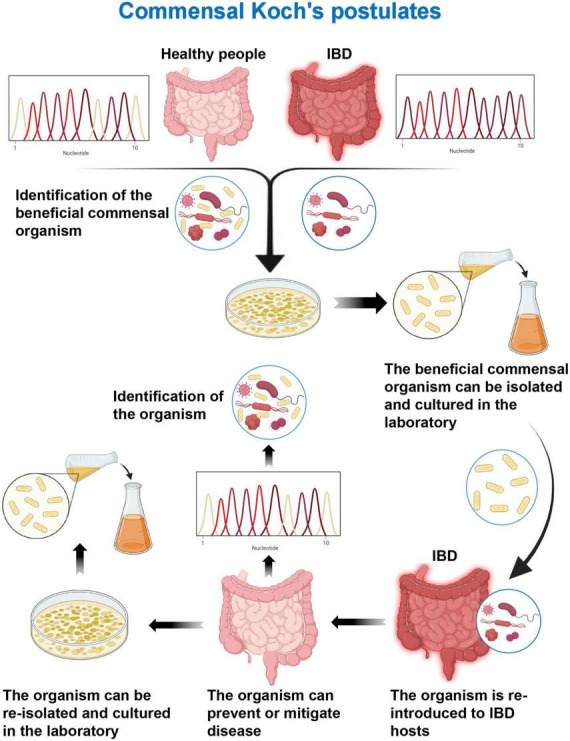
Commensal Koch’s postulates. Detection of health-related specific beneficial commensal microorganisms by sequencing in healthy individuals and inflammatory bowel disease (IBD) patients. The beneficial commensal organism can be isolated and cultured in the laboratory. Then the organism can prevent or mitigate disease when it is re-introduced to IBD hosts. The commensal organism can be detected and re-isolated from healthy recovered hosts.

Koch’s postulates of symbiotic microorganisms (one beneficial microorganism, one improvement in disease state): (1) commensal strains are associated with host health and are regularly found in healthy hosts but less frequently in diseased hosts; (2) the symbiotic bacteria can be isolated as cultures and grown in the laboratory; (3) when the symbiotic strain is introduced into a new host, it can ameliorate or alleviate symptoms; and (4) when the symbiotic strain is introduced into a restored host, this symbiotic strain would be detected (Figure 3) (90). The symbiotic microorganism rule is used to supplement the symbiotic microorganisms of IBD patients to restore the balance of the intestinal microflora, so as to achieve the improvement of the disease state or the remission of clinical symptoms, considering the role of dysbiosis in the pathogenesis of IBD with reverse thinking. It has been reported that two species of Lactobacillus and Bifidobacterium play important physiological functions in healthy individuals (33, 34, 91), while their numbers are significantly reduced in IBD patients (32). Some researchers have isolated two genera Lactobacillus and Bifidobacterium from the feces of healthy mice, and the isolated genera have been cultured, and identified by the Gram’s method. The cultured genera can tolerate simulated gastrointestinal conditions. The two types of bacteria are combined, and called Personalized Probiotic Mix (PP). The experiment has used Dextran Sulfate Sodium Salt to induce colitis mouse model for control experiments. Mice with DDS-induced colitis orally administered PP have less weight loss, lower disease activity index, and fewer clinical signs of disease (hunched back, less movement, and stray hair) compared to controls. In addition, PP can more effectively modulate the host immune response, reduce the expression of pro-inflammatory factors (IL-1β and IL-6), and increase the expression of anti-inflammatory factors (TGF-β and IL-10) (92). The genus Bacteroides has important physiological functions in the gut microflora of healthy individuals, whereas patients with IBD have a reduced number of commensal anaerobic bacteria, including members of the genus Bacteroides. Delday et al. (93) have evaluated the effect of Bacteroides polymorpha, an important component of the Bacteroides genus, on colitis using DDS-induced and IL-10 knockout IBD mouse models, respectively. In the DDS model experiment, compared with the control group (gavage with DDS alone), rats received with gavage of Bacteroides polymorpha showed significant improvement in weight loss, with significantly lower levels of colon histopathological scores and inflammation-related genes (such as IL-6 and IL-1b). The expression of tumor necrosis factor alpha is significantly downregulated. In the IL-10 knockout model, compared with the control group (receiving culture medium alone), the rats in the experimental group had significantly increased body weight, and lower histopathological scores, while macroscopically, colonic edema and tissue destruction were extensive. Inflammatory infiltration was significantly improved, and the expression levels of pro-inflammatory genes (such as Arg1, I-L6, Ccl3, Spp1, and I-L1a) were significantly decreased (93). This suggests that the microbiota transplantation of healthy individuals can restore the balance of intestinal microflora by supplementing the commensal microbiota, destabilizing the antibacterial flora, and reducing the inflammatory response of the intestinal tract, which is in line with the first three items of the Koch’s postulates of commensal microbes. However, further researches are still needed to complement the existing experiments.

## Summary and outlook

Human microbial macrogenomic DNA sequencing has revealed the diversity and complexity of the composition of the human gut microbiota, which is essentially a highly complex community almost indistinguishable from other ecological communities in the natural environment. As hosts, humans coexist and co-evolve with the gut microbiota, maintaining a delicate balance. The stability of the host-gut microbial ecosystem plays a crucial role in human health. In IBD patients, the delicate host-microbiota balance has been disrupted, with significantly altered composition of the gut microbiota, reduced diversity, and altered ratios of pathogenic microbes to symbiotic microbes, resulting in microbiota dysbiosis. This dysbiosis will not only lead to changes in the metabolic pathways of related microorganisms, but also be associated with abnormal immune responses, weakened intestinal barrier function, and genetic susceptibility to diseases. However, the causal relationship of gut microbiota dysbiosis and IBD has not been fully elucidated. Meanwhile, with the progress of research on the role of dysbiosis in the occurrence and development of IBD, the therapeutic effect of microbial preparations in IBD has gradually been paid attention to, and it is considered to be an effective and promising method for the treatment of IBD. Antibiotics, prebiotics, probiotics, and FMT have made encouraging progresses in the treatment of IBD. However, the optimal treatment strategies regarding the optimization of the administration route, treatment time and frequency, and, appropriate strain selection, as well as reducing adverse reactions, still face significant challenges.

Nowadays, the era of whole-genome sequencing has been coming, and further revisions to disease causality guidelines are necessary, especially for the complex diseases such as IBD, where the Koch’s postulates needs to be extended to accommodate polymicrobial triggers. The modified Koch’s postulates is still valuable in proving causality. This article utilizes two extended Koch’s postulates, by reviewing some experimental results, from forward (ecological Koch’s postulates, i.e., the microbial dysbiosis can transmit disease) and reverse (symbiotic microbial Koch’s postulates, i.e., improving dysregulation to re-establish balance can alleviate the disease) directions, expounding the important position of dysbiosis in the pathogenesis of IBD. The current challenge is that the existing experiments do not meet the full conditions of the rule and need to be supplemented by further researches. Meanwhile, given the recent understanding of microbial populations, we should consider them in the broader context of system and etiology of disease, including the host’s genetic susceptibility, abnormal immune response, health status, and microflora. With the advancement of genome sequencing technology and the identification of new host-microbe interaction mechanisms, we need to further expand and supplement the Koch’s postulates to meet its application in non-infectious and complex modern diseases. By linking the modern Koch’s postulates to the microbiome, it is expected not only to reveal the potential causal relationship between microbial dysbiosis and IBD, but also to guide the development of optimized microbial preparations to provide more effective treatment for IBD patients.

## Author contributions

HZ devised concept of manuscript. HZ and WZ wrote the manuscript. YL and YH contributed to creation of the figures and made significant revisions. LY and DC contributed to discussions about the manuscript. All authors contributed to the article and approved the submitted version.
